# Well-being, physical activity and health promotion among officers and ratings on board cargo ships: A cross-sectional study

**DOI:** 10.1371/journal.pone.0333588

**Published:** 2025-09-30

**Authors:** Chiara Reck, Dorothee Dengler, Lukas Belz, Volker Harth, Marcus Oldenburg

**Affiliations:** Department of Maritime Medicine, Institute for Occupational and Maritime Medicine (ZfAM), University Medical Center Hamburg-Eppendorf, Hamburg, Germany; Vietnam Maritime University, VIET NAM

## Abstract

**Background:**

Health and well-being of seafarers working on merchant vessels is an essential area of research, given the significant physical and psychological demands placed on these workers. The present study focuses on assessing the current health status and well-being, identifying personal needs and requirements for health promotion among officers and ratings on board.

**Methods:**

A total of 583 seafarers from a German shipping company were surveyed by using a questionnaire including the standardized WHO-5-Well-being Index, PHQ-9, GPAQ and questions about the need for health measures as well as physical activity.

**Results:**

The survey was distributed to 616 seafarers on 33 merchant ships with a response rate of 94.6%. Officers reported lower well-being compared to ratings (60.1% vs. 70.0%, p = 0.001). Regarding depressive symptoms no significant differences between the rank groups were found. Furthermore, ratings stated a significantly higher total physical activity than officers (10,437 vs. 8,035 MET-min/week, p = 0.005), while officers were significantly more sedentary compared to ratings (3.5 vs. 2.4 hours/day, p = 0.001). Seafarers also reported a high interest in sports on board (>56%) and health promotion (>60%), with a preference for digital formats and monetary incentives for participation in health programs.

**Conclusion:**

The impaired well-being of seafarers compared to shore-based reference groups underscores the need for targeted, digital interventions to improve the health and well-being of seafarers. The findings highlight significant differences in well-being, physical activity, and interest in health promotion between officers and ratings. Future research should apply longitudinal designs to better understand the causal relationships between occupational and socio-demographic factors with health outcomes among seafarers.

## Background

Seafaring on cargo vessels is characterized by extended periods of isolation, irregular working hours, and high occupational demands, which can significantly challenge the well-being of crew members [1]. However, the distribution of occupational stressors experienced on board are not equal across ranks. Officers, who typically assume higher responsibility for navigation and ship management, often face cognitive strain associated with decision-making under pressure and the burden of command. In contrast, ratings are more frequently exposed to physical labor, repetitive tasks, and reduced autonomy, which can lead to distinct forms of mental and physical exhaustion. Beyond that, the rigid occupational hierarchy, while essential for safety and operational efficiency, can hinder personal development and self-realization, especially among lower-ranked crew members with limited career advancement opportunities [2,3].

Over and beyond, seafarers frequently experience psychosocial stress due to limited contact with their families and friends, cultural and linguistic diversity on board, and long durations away from home [3]. Furthermore, seafarers are frequently affected by reduced sleep duration and impaired sleep quality due to irregular watch systems and high workloads as well as responsibilities [4,5]. Environmental conditions – such as noise, ship movement, and vibration – can impact sleep quality across all ranks, but the effects may be more pronounced among ratings due to their less favorable sleeping quarters, situated closer to the engine [6]. Insufficient sleep is also strongly linked to decreased psychological well-being, contributing to increased levels of stress, depressive symptoms, and emotional exhaustion [7].

Well-being, as defined by the World Health Organization (WHO), includes not only the absence of disease or infirmity, but a state of complete physical, mental, and social well-being [8]. When applied to the maritime context, this definition underscores the necessity of addressing both psychological and environmental stressors to ensure the holistic well-being of officers and ratings. The overall rates of depression and suicidal tendencies are described to be higher compared to land-based populations [9]. This highlights the need for targeted mental health interventions for seafarers, particularly those in higher-ranked positions who may be at greater risk due to high responsibilities and mental strain [9].

Physical activity is a critical determinant of health, with established benefits for both physical and mental well-being [10,11]. Among seafarers, however, opportunities for recreational physical activity are often limited due to spatial constraints, unpredictable work schedules, and exhaustion due to physically and mentally demanding tasks [1]. Occupational physical activity of ratings on cargo ships typically involves manual labor and maintenance tasks, officers face tasks like brisk walking up- and downstairs and watchkeeping duties. Therefore, seafarers generally meet or exceed the WHO’s minimum physical activity requirements, solely due to work-related physical activity [12]. But the health benefits of occupational physical activity are debated. Some studies show positive effects [13,14], while others report no effect [15] or negative effects on cardiovascular diseases and mortality [16,17]. In contrast, the health benefits of leisure-time physical activity and sports are more consistently supported. These inconsistencies may be partly explained by the varying nature of occupational tasks [18]. A 2008 study by Abu-Omar and Ruetten found that leisure-time activity is linked to better self-rated health [19].

The potential challenges related to lack of motivation and barriers to being active can be addressed through measures aimed at enhancing the attractiveness of doing sports [20]. Beyond that, fatigue, mentally as well as physically, diminishes motivation and energy, reducing the likelihood for participation in leisure activities, including physical exercises [21]. However, people who regularly participate in leisure-time physical activity report improved sleep quality, reduced stress levels, and enhanced mood [22,23].

Given the multidimensional health challenges faced by officers and ratings, the implementation of tailored health promotion strategies is essential. Effective approaches should address both occupational and lifestyle-related health risks, incorporating elements of education, infrastructure, and policy.

On the organizational level, the provision of dedicated recreational facilities, ergonomic work environments, and healthy dietary options are fundamental to creating health-promoting conditions on board. A Danish study showed that implemented health promotion programs had a positive impact on seafarer’s health behavior, such as sugar intake and physical fitness [24]. Psychosocial interventions, including stress management training can foster participants’ mental health and resilience [25].

Despite indications of differing stress patterns, a clear comparative analysis of health-promoting measures or preventive strategies for officers versus ratings is still lacking. The present study therefore focuses on assessing the current health status and well-being, identifying personal needs and requirements for health promotion among both officers and ratings on board. This perspective, which has so far received only limited attention, is essential to deepen our understanding of rank-specific well-being patterns as well as to develop customized health promotion programs that are tailored to the unique occupational demands of seafaring life.

## Methods

The study was based on a cross-sectional questionnaire survey of an international sample of seafarers working on merchant vessels of one shipping company from Hamburg, Germany. The survey, utilizing questionnaires in English, was implemented to assess the current health status, identify personal needs and requirements, and gather opinions on specific health-related topics among the employees onboard. All 616 seafarers on 33 merchant ships, without exclusion criteria to reflect the full range of experiences and needs, were asked to participate in the survey conducted from February 11, 2022 to February 21, 2022. The electronic questionnaire was emailed to the captains, printed, forwarded to the crew members onboard, collected within two weeks, scanned, and then returned by email to the researchers, without any involvement from land-based company personnel. The data analysis was conducted via IBM SPSS Statistics 29.

Initially the participants were informed in writing that their data would be processed confidentially and anonymously, and that their participation was voluntary. All participants provided verbal informed consent prior to being included in the study. Due to the anonymous nature of the study, no documentation of consent was retained for individual participants. Crew members verbally expressed their willingness to participate to their respective captains, who then distributed the questionnaires. The responsible ethics committee, the Hamburg Medical Chamber (ger.: Ethik-Kommission der Hamburger Ärztekammer), reviewed and approved this procedure, including the verbal consent process and the maintenance of participant anonymity (PV7174).

Out of a total of 616 distributed surveys, 583 (94.6%) returned. The questionnaires main topics were well-being and mental health (15 questions) and sport on board (35 questions), furthermore, the seafarers were asked about their interest in health promotion on board (4 questions).

### Well-being

#### WHO-5 wellbeing-index.

The WHO-5 Well-Being Index is a standardized five-item questionnaire measuring subjective well-being [26]. Each item is rated from 0 to 5, yielding a total score of 0–25, which is then converted to a standardized percentage score, ranging from 0–100%, by multiplying the total score by 4. Higher scores indicate better well-being, and a score below 50% suggests possible depression [26]. Other studies have given Cronbach’s alpha values ranging from 0.81 to 0.92 [27,28]. These values suggest that the questionnaire has high internal consistency.

### Psychological health

#### Patient health questionnaire (PHQ-9).

The Patient Health Questionnaire-9 (PHQ-9) was employed to assess depressive symptoms. It is based on the diagnostic criteria for major depressive disorders outlined in the Diagnostic and Statistical Manual of Mental Disorders, Fifth Edition (DSM-5) [29]. It consists of nine items, each corresponding to one of the core symptoms of depression. Each item is scored on a four-point Likert scale ranging from 0 (“not at all”) to 3 (“nearly every day”), resulting in a total score between 0 and 27. Higher scores indicate greater severity of depressive symptoms. Total scores were categorized as follows: 0−4 (none-minimal), 5−9 (mild), 10−14 (moderate), 15−19 (moderately severe), and 20−27 (severe). In accordance with established cut-off values a value of 10 or higher is indicative of a depressive tendency [29]. The PHQ-9 has demonstrated good reliability and validity in diverse populations and is widely used in epidemiological and clinical research [30]. Research has demonstrated that the PHQ-9 possesses high internal consistency, with a Cronbach’s alpha of 0.84 [31].

### Sport on board

#### Global physical activity questionnaire (GPAQ).

The Global Physical Activity Questionnaire (GPAQ), developed by the World Health Organization (WHO), is used to assess physical activity behavior [32]. The GPAQ captures information on physical activity in three domains: activity at work, travel to and from places and recreational activities. It also includes a question on sedentary behavior. The examples listed in the questionnaire were adapted to the maritime context in each category (e.g., lashing, maintenance or walking while shore leaves). Based on the reported activity information, total physical activity was calculated in “metabolic equivalent of task minutes” per week (MET-min/week), following WHO guidelines [32]. The metabolic equivalent of task (MET) quantifies the energy expenditure of physical activities relative to the resting metabolic rate, with 1 MET representing rest, i.e., approximately 1 kcal per kg of body weight per hour.

MET-minutes are calculated by multiplying the MET value of an activity by its duration in minutes. The World Health Organization recommends a minimum of 600 MET-min/week– equivalent to 150 minutes of moderate (= 4 MET) or 75 minutes of vigorous (= 8 MET) activity – to achieve substantial health benefits [12]. According to the Global Burden of Disease (GBD) 2013 study, physical activity is divided into categories: Inactivity (less than 600 MET-min/week), low activity (600–3,999 MET-min/week), moderate activity (4,000–7,999 MET-min/week) and high activity (more than 8,000 MET-min/week) [33].

The GPAQ Analysis Guide [32] was used for data processing and cleansing to ensure consistency and comparability with international data standards. Based on these criteria, a total of 565 cases were assessed for data validity. After applying the recommended cleaning procedure (to exclude non plausible data (e.g., rigorous activities > 16 hours per day), 492 cases (87.1%) were classified as valid and retained for further analysis, while 73 cases (12.9%) were excluded due to invalid activity data.

In contrast to the WHO-5 and PHQ-9, no established Cronbach’s alpha values are available for the GPAQ. However, the GPAQ demonstrated moderate to substantial reliability (Kappa 0.67 to 0.73; Spearman’s rho 0.67 to 0.81) and moderate to strong concurrent validity (range 0.45 to 0.65) with the International Physical Activity Questionnaire (IPAQ), an already validated and widely used instrument for detecting overall physical activity through self-assessment [34]. The GPAQ was selected because it focuses on the distinction between work-related and leisure-time physical activity, which was considered particularly relevant for seafarers in the context of this study.

### Type of sports

To assess interests in sport activities on board, four self-developed items were designed by a multidisciplinary team comprising a sports scientist, occupational physician, a general practitioner, and a nautical expert. These items addressed preferences for sport competitions among crew members are queried: group as well as individual sports, interest in exercise instructions and monitoring training progress. The items are based on a five-point Likert scale ranging from “Strongly disagree” to “Strongly agree”, with an additional option “Don’t know”.

### Barriers to being active quiz

The present study used a version of the Centers for Disease Control and Prevention’s (CDC) quiz about barriers to physical activity adapted to the maritime environment to collect data on participants’ perceived reasons for not exercising. The quiz includes questions on common barriers such as lack of time, motivation, social influences and access to facilities [35]. The responses were analyzed to identify common patterns and examine associations between specific barriers, such as rank-related, demographic or behavioral variables.

### Interest in health promotion on board

To assess health-related interests and preferences, questions about interest in the health topics physical activity, nutrition, relaxation and fatigue are asked. These topics were shown to be the most important and interesting for seafarers in a previous study [36]. Moreover, up to two preferred health program options, such as health officer-led sessions or health apps, as well as up to two preferred incentives for participating in health promotion programs (e.g., participation during working hours or mobile phone credit) are to be selected.

### Statistics

Statistical analysis was performed using IBM SPSS Statistics 29, with a significance threshold set at α = 5% (p ≤ 0.05). Descriptive statistics were calculated to summarize the distribution of responses. Group differences involving dichotomous variables were examined using the Pearson Chi-square test. Odds ratios (OR) with 95% confidence intervals (CI) were computed via binary logistic regression for significant group differences. Adjusted odds ratios were calculated by including relevant covariates such as age and culture in the regression models. The t-test for independent samples was applied to examine differences between groups for metric variables. Additionally, multiple linear regression was employed to investigate the relationship between multiple independent variables and a continuous dependent variable to evaluate the combined effect of several predictors while adjusting for potential confounding factors.

## Results

### Demographic and occupational data

Of a total of 583 seafarers, 552 (97.7%) provided information about their gender. Among the study group, 95.3% (n = 526) identified as male, whereas 3.8% (n = 21) and 0.9% (n = 5) identified as female or did not specify their gender. The majority of participants (n = 374 (67.2%)) was younger than 40 years. 234 (43.1%) were from Europe, 246 (45.6%) from Asia and 60 (11.1%) from America.

565 (96.9%) participants reported about their rank on board. 215 (38.1%) were officers and 350 (61.9%) were ratings. 18 persons did not provide any information on their rank and therefore were excluded from further analyses. From the 565 included participants 285 (50.4%) had watch keeping duties, related to the rank indicated. Crew members were more likely to be officers when coming from Europe (66%) than from a non-European country (34%). On the other hand, ratings were more often non-Europeans (71.3%) than Europeans (28.7%). Overall, the entire cohort was composed of 43.1% Europeans and 56.9% non-Europeans. Detailed demographic data are displayed in Table 1.

**Table 1:** Demographic data of occupational groups.

Characteristic	Total* (n = 565)	Officers* (n = 215)	Ratings* (n = 350)
**Gender**, n (%)			
Male	526 (95.3)	197 (93.8)	329 (96.2)
Female	21 (3.8)	11 (5.2)	10 (2.9)
Not Specified	5 (0.9)	2 (1.0)	3 (0.9)
**Age**, n (%)			
29 years or younger	163 (29.3)	35 (16.5)	128 (37.1)
30–39 years	211 (37.9)	108 (50.9)	103 (29.9)
40–49 years	96 (17.2)	40 (18.9)	56 (16.2)
50–59 years	72 (12.9)	21 (9.9)	51 (14.8)
60 years or older	15 (2.7)	8 (3.8)	7 (2.0)
**Culture**, n (%)			
European	234 (43.3)	138 (66.0)	96 (29.0)
Asian	246 (45.6)	51 (24.4)	195 (58.9)
American	60 (11.1)	20 (9.6)	40 (12.1)
**Work area**, n (%)			
Deck	320 (58.0)	112 (52.1)	208 (61.7)
Engine	188 (34.0)	103 (47.9)	85 (25.2)
Galley	44 (8.0)	---	44 (13.1)
**Watch keeping duties**, n (%)	285 (50.4)	112 (52.1)	173 (49.4)
**Years as a seafarer**, mean (SD)	12.6 (9.5)	14.5 (9.2)	11.4 (9.5)
**BMI**, mean (SD)	25.9 (3.4)	26.6 (3.3)	25.5 (3.4)

* The quantity of analyzed responses varies per question dimension, diverging from the main population.

### Body-mass-index

The Body-Mass-Index (BMI) was on average 25.9 (SD 3.4). Based on the Pearson-Chi-Square-Test a significant association was found between BMI category and rank on board (p = 0.011). Ratings were more frequently classified as normal weight (BMI < 25; 48.2%) compared to officers (35.4%). Individuals classified as overweight (BMI 25–30) or obese (BMI > 30) accounted for 48.1% and 16.5% of the officers, respectively, and 39.7% and 12.1% of the ratings.

### Well-being

#### WHO-5-Well-being Index and Patient Health Questionnaire (PHQ-9).

In the sample of 557 seafarers, significant differences in subjective well-being were observed between the professional groups. Officers reported a significantly lower mean score of 60.1% (SD 19.8), compared to 70.0% (SD 19.7) among ratings (p < 0.001).

Low well-being, defined as a WHO-5 score below 50%, was present in 19.7% of the overall sample (Table 2). A markedly higher proportion of officers (27.1%) reported low well-being compared to ratings (15.2%) (p < 0.001). The crude odds ratio (OR) for low well-being among officers compared to ratings was 2.08 (95% CI 1.37–3.17). This association remained statistically significant after adjusting for the confounders culture (Europeans vs. Non-Europeans) and age (younger than 40 years or 40 years and older) (aOR 1.98 (95% CI 1.24–3.16)). These findings indicate that officers are at a significantly increased risk of reduced psychological well-being compared to ratings.

**Table 2:** Results WHO-5-Wellbeing-Index and Patient Health Questionnaire (PHQ-9).

	Total (n = 557)	Officers (n = 214)	Ratings (n = 343)	P*
**WHO-5-%-Score**, mean (SD)	66.2 (20.3)	60.1 (19.8)	70.0 (19.7)	**0.001** ^$^
**Low Well-being**, n (%)	110 (19.7)	58 (27.1)	52 (15.2)	**0.001** ^**&**^
**PHQ-9 Score**, mean (SD)	4.4 (4.2)	4.1 (3.9)	4.5 (4.5)	0.210^$^
**PHQ-9 Depression**^**a**^, n (%)	59 (10.6)	22 (10.2)	37 (10.8)	0.836^**&**^
**Suicidal tendencies**^**b**^, n (%)	50 (8.9)	37 (10.8)	13 (6.0)	0.058^**&**^

* Significant results are printed in bold.

^$^ T-Test for independent samples.

^&^ Chi-square-test.

^a^ Depression = PHQ-9-Score ≥10.

^b^ PHQ-9 Item No. 9 “thoughts about better being of dead or self-hurting thoughts” was answered with “several days” or more.

In contrast, no significant differences were found in depressive symptoms as measured by the PHQ-9, as shown in Table 2. 50 (8.9%) participants reported suicidal tendencies by thinking about hurting themselves or wishing to be dead for at least several days in the past two weeks.

### Physical activity

#### Global physical activity questionnaire (GPAQ).

In the sample, participants reported a weekly average of 9,436 MET-minutes of physical activity, with ratings showing significantly higher activity levels than officers (p = 0.005). This difference was primarily driven by work-related activity, where ratings reported higher values (p = 0.001). Overall sedentary behavior averaged 2.8 hours per day but differed markedly by rank: officers reported more sedentary time than ratings (p = 0.001) (Table 3).

**Table 3:** Physical activity on board.

	Total (n = 492)	Officers (n = 205)	Ratings (n = 287)	p*^$^
**Total activity/ week,** mean MET-min (SD)	9,436 (9,438)	8,035 (8,494)	10,437 (9,952)	**0.005**
**Work activity/ week**, mean MET-min (SD)	7,027 (8,718)	5,534 (7,407)	8,094 (9,412)	**0.001**
**Leisure activity/ week**, mean MET-min (SD)	1,604 (2,924)	1,703 (3,003)	1,533 (2,870)	0.391
**Travel activity/ week**, mean MET-min (SD)	805 (1,780)	797 (1,693)	810 (1,842)	0.801
**Sedentary behavior/ day**, mean hours per day (SD)	2.8 (2.6)	3.5 (2.8)	2.4 (2.3)	**0.001**

*Significant results are printed in bold.

^$^ T-test for independent samples.

Multiple linear regression analyses were conducted to assess the extent to which rank (officers vs. ratings), age category, and culture (Europeans vs. Non-Europeans) predict physical activity levels in the domain’s activity during work and leisure time, measured in MET-min/week. 471 participants were included in the analyses.

1. Physical activity during working hours per week
  The regression analysis showed that the model explained 2.2% of the variance in physical activity during work hours, with a significant overall effect (F = 3; 581, p = 0.014). Ratings (B = 2804, p = 0.001) reported significantly more physical activity than officers. Age and culture had no significant impact (p > 0.05). The model’s explanatory power was limited.
2. Leisure time physical activity per week
  Multiple regression analysis showed that the model explaining leisure time physical activity (MET-min/week) was not significant, with an R-squared value of 0.014, indicating that the independent variables explained only 1.4% of the variance.

### Sport on board

A high proportion of both groups, officers (80.9%) and ratings (77.4%), expressed interest in sport competitions. This difference was statistically significant (p = 0.023), but not confirmed by adjusted logistic regression (aOR=0.71, 95% CI 0.41–1.25). Similarly high levels of interest were found for group activities (66.0% vs. 63.7%) and individual sports (60.9% vs. 56.6%), with no significant differences between ranks. Around 70% of both groups reported interest in exercise instruction and monitoring (Table 4).

**Table 4:** Interest in sport on board.

	Total (n = 565)	Officers (n = 215)	Ratings (n = 350)	p*^&^	Crude OR*^1^ (95% CI)	Adj. OR*^1,2^ (95% CI)
**Interest in …**						
**… sport competitions**, n (%)	445 (78.8)	174 (80.9)	271 (77.4)	**0.023**	**0.56 (0.33–0.96)**	0.71 (0.41–1.25)
**… group sport activities**, n (%)	365 (64.7)	142 (66.0)	223 (63.7)	0.832	0.66–1.40	0.96 (0.66–1.40)
**… individual sport**, n (%)	329 (58.2)	131 (60.9)	198 (56.6)	0.652	0.92 (0.64–1.33)	1.15 (0.77–1.74)
**… exercise instruction and monitoring**, n (%)	397 (70.3)	150 (69.8)	247 (70.6)	0.627	1.12 (0.72–1.74)	1.06 (0.65–1.72)

All percentages refer to the number of seafarers who answered each question.

* Significant findings are printed in bold.

^&^ Chi-square-test.

^1^Officers in comparison to ratings.

^2^Adjusted for age and culture groupNo significant association was observed between rank and the preference for individual sports. However, age showed a significant effect: older participants were more likely to prefer individual sports (p = 0.016).

Several reasons have been identified that prevent seafarers from being more physically active, as shown in Table 5. The prevalence of time-related barriers that decrease activity was observed to be significant: 70.8% of the total sample reported that their day is too busy, with no statistically significant difference between the ranks. Fatigue-related barriers were also common, with 60.7% of the total sample feeling too tired after work and 45.8% reporting not enough sleep, without significant group differences.

**Table 5:** Barriers to being active.

What keeps you from being more active, n (%)	Total (n = 565)	Officers (n = 215)	Ratings (n = 350)	p*^&^	Crude OR*^1^ (95% CI)	Adj. OR*^1,2^ (95% CI)
**Lack of time**						
Day is too busy	400 (70.8)	157 (73.0)	243 (69.4)	0.362	0.84 (0.58–1.23)	0.80 (0.53–1.21)
Physical activity is too time-consuming	223 (39.5)	66 (30.7)	157 (44.9)	**0.001**	**1.84 (1.28–2.63)**	**1.78 (1.20–2.63)**
Free time is too short	304 (53.8)	108 (50.2)	196 (56.0)	0.182	1.26 (0.90–1.77)	1.14 (0.78–1.65)
**Social influence**						
Embarrassment	121 (21.4)	34 (15.8)	87 (24.9)	**0.011**	**1.76 (1.14–2.73)**	1.56 (0.96–2.53)
**Lack of energy**						
Too tired after work	343 (60.7)	136 (63.3)	207 (59.1)	0.331	0.84 (0.59–1.19)	0.79 (0.54–1.17)
Not enough sleep	259 (45.8)	94 (43.7)	165 (47.1)	0.428	1.15 (0.82–1.62)	1.01 (0.69–1.46)
**Lack of motivation**						
Troubles of getting started to exercise	316 (55.9)	108 (50.2)	208 (59.4)	**0.033**	**1.45 (1.03–2.04)**	1.41 (0.97–2.06)
Finding excuses is easier	186 (32.9)	61 (28.4)	125 (35.7)	0.071	1.40 (0.97–2.03)	1.35 (0.90–2.02)
Difficulties in continuing to exercise	252 (44.6)	85 (39.5)	167 (47.7)	0.058	1.40 (0.99–1.97)	1.27 (0.87–1.86)
**Fear of injury**						
People hurt themselves	155 (27.4)	35 (16.3)	120 (34.3)	**0.001**	**2.68 (1.76–4.10)**	**2.26 (1.42–3.61)**
Afraid of injury or heart attack	92 (16.3)	20 (9.3)	72 (20.6)	**0.001**	**2.56 (1.49–4.28)**	**1.91 (1.09–3.36)**
**Lack of skill**						
Missing skills	116 (20.5)	25 (11.6)	91 (26.0)	**0.001**	**2.67 (1.65–4.32)**	**2.34 (1.38–3.97)**
Not good enough for having fun	108 (19.1)	34 (15.8)	74 (21.1)	0.118	1.43 (0.91–2.23)	1.18 (0.72–1.92)
**Physical demanding work**	337 (59.6)	105 (48.8)	232 (66.3)	**0.001**	**2.06 (1.46–2.92)**	**2.03 (1.38–2.98)**

All percentages refer to the number of seafarers who answered each question.

* Significant findings are printed in bold.

^&^ Chi-square-test

^1^Officers in comparison to ratings

^2^Adjusted for age and culture group

Fear-related barriers were more prevalent in ratings: significantly more reported fear of injury or heart attack (20.6% vs. 9.3%) and concerns that people hurt themselves during exercise (34.3% vs. 16.3%). Ratings also more often reported a lack of skills (26.0% vs. 11.6%). Physically demanding work was reported as a barrier to physical activity by a substantial portion of the sample (59.6%). This barrier was also significantly more prevalent among ratings than among officers (66.3% vs. 48.8%).

### Interest in health promotion on board

Descriptive analyses were conducted for participants’ interest levels across four domains of health-related information: physical activity, nutrition, relaxation, and fatigue. Mean interest scores revealed the highest average interest in nutrition (3.3 (SD 1.7)), followed by physical activity (3.1 (SD 1.7)), relaxation (3.0 (SD 1.7)), and fatigue (2.8 (SD 1.8)).

Independent samples t-tests were conducted to examine differences in health-related information interest between officers and ratings. Significant differences were observed across all four domains: ratings reported consistently significantly higher interest in information about health-related topics compared to officers. The exact findings are shown in the Table 6 below.

**Table 6:** Interest in health topics by rank.

Information Topic	Officers	Ratings	p*^&^
Physical activity, mean (SD)	2.9 (1.8)	3.2 (1.7)	**0.048**
Nutrition, mean (SD)	3.0 (1.8)	3.5 (1.6)	**0.001**
Relaxation, mean (SD)	2.7 (1.8)	3.1 (1.7)	**0.012**
Fatigue, mean (SD)	2.5 (1.8)	3.0 (1.7)	**0.004**

* Significant results are in bold.

^&^ T-test for independent samples.

Multiple linear regressions were performed to assess the impact of rank (officers vs. ratings), age (categorized), and culture (European vs. Non-European) on participants’ interest (Table 7).

**Table 7:** Summary of multiple regression analysis, interest in health topics.

Dependent variable	Indipendent variable	B	β	P*	95% CI (B)*
**Physical activity**	Rank¹	0.142	0.041	0.386	(−0.180, 0.465)
	Age²	−0.502	−0.138	**0.002**	**(−0.822, −0.182)**
	Culture³	0.443	0.130	**0.008**	**(0.119, 0.767)**
**Nutrition**	Rank¹	0.320	0.092	0.051	(−0.002, 0.641)
	Age²	−0.501	−0.137	**0.002**	**(−0.817, −0.184)**
	Culture³	0.493	0.144	**0.003**	**(0.173, 0.813)**
**Relaxation**	Rank¹	0.146	0.042	0.363	(−0.169, 0.461)
	Age²	−0.315	−0.086	**0.050**	**(−0.630, 0.000)**
	Culture³	0.736	0.213	**0.001**	**(0.420, 1.053)**
**Fatigue**	Rank¹	0.344	0.095	**0.041**	**(0.014, 0.675)**
	Age²	−0.293	−0.077	0.081	(−0.622, 0.037)
	Culture³	0.380	0.107	**0.025**	**(0.049, 0.711)**

*Significant results are printed in bold.

^1^ Rank is categorized in 1 = Officers, 2 = Ratings, higher values correspond to ratings

^2^Categorized age (1 = younger than 40, 2 = 40 years or older), higher values correspond to older age

^3^Culture is categorized in 1 = Europe, 2 = Non-Europe, higher values correspond to Non-Europe

For physical activity, the model was significant (F (3; 492)=8.41, p = 0.001) with an adjusted R^2^ of 0.043, indicating that approximately 4.3% of the variance in interest in physical activity was explained by the model. Interest was significantly lower among older participants (p = 0.002) and higher among individuals from Non-European regions (p = 0.008). Rank had no significant effect.

For nutrition, the model explained more variance (F (3; 496)=11.50, p = 0.001, adj. R^2^ = 0.059). Age younger than 40 years (p = 0.002) and Non-European culture (p = 0.003) were significant predictors for a higher interest. The effect of rank was marginal (p = 0.051), but there was a tendency that ratings are more interested in information about nutrition.

In terms of relaxation, the model was significant (F (3; 517)=12.57, p = 0.001, adj. R^2^ = 0.063). A strong positive effect of Non-European culture (p = 0.001) and a negative effect of age (p = 0.050) were found. The rank showed no significant association.

For fatigue, the model explained variance of 3.1% (F (3; 514)=6.54, p = 0.001, adj. R^2^ = 0.031). Significant positive effects were observed for ratings (p = 0.041) and Non-Europeans (p = 0.025), while the negative effect of age was not statistically significant (p = 0.081).

Across all models, age and culture were the most consistent predictors, whereas rank had a weaker and less consistent influence.

Finally, participants were asked to indicate which types of health-related program elements they would prefer on board. Across all 547 valid cases, digital formats were generally preferred over analog materials. Apps were selected most frequently (42.0%), followed by quiz games and competitions (35.5%) and regular teaching by the “health officer” (26.1%). In contrast, specific health information flyers for seafarers (13.7%) was least frequently selected.

When asking about preferred incentives for participation in a health promotion program on board, the majority of respondents (n = 552) showed a clear preference for monetary compensation in the form of mobile phone credit (56.3%). Other incentives, such as more free time on board (35.5%) or a barbecue (29.3%), were selected also frequently, while options like receiving a fitness tracker (19.2%) or doing a health program during work hours (18.5%) were least favored.

## Discussion

The findings of this study highlight several critical aspects of the health status, well-being and physical activity of seafarers on merchant vessels and indicate significant differences between officers and ratings, as well as between different demographic groups.

### Mental health status of seafarers

With a WHO-5-Well-Being Index of 66.2%, the findings of the present study are similar to the findings of 69.7% from a recent pilot study on digitally supported health promotion for seafarers [36]. The WHO-5 revealed that officers had a nearly twofold increased risk for low well-being compared to ratings. These findings are consistent with previous research indicating that higher-ranked seafarers often have decreased well-being and impaired mental health. The authors concluded that this is due to the job-related particularly high stress and responsibility of this occupational group [1].

In contrast, the PHQ-9 score did not show significant differences in severity of depressive symptoms between officers and ratings. This suggests that while well-being may differ, the prevalence of depressive symptoms is relatively similar across rank groups. In comparison to a land-based group that is representative for the German population the proportion of those on board who suffer from at least moderate depressive symptoms is almost twice as high (5.6% vs. 10.6%) [37].

Based on PHQ-9, the Seafarer Mental Health Study [38], published in October 2019, observed in 25% of seafarers’ indicators for at least moderate depression and in 20% signs for suicidal ideation, while the present sample appears to exhibit distinctly lower levels of both depressive symptoms (10.6%) and suicidal thoughts (8.9%). This discrepancy may reflect differences in occupational environments or mental health support systems available to the respective populations.

However, the presence of suicidal tendencies in 8.9% of participants is very concerning and warrants further attention and intervention. Even though the difference in suicidal tendencies between officers and ratings is not statistically significant (p = 0.058), it is a notable tendency for officers to exhibit higher levels of suicidal ideation compared to ratings (10.8% vs. 6.0%). This assumption is confirmed by the findings of the German mortality study on deaths of seafarers at sea [9]. The study revealed that all of the five documented cases of suicide from 1998 to 2008 were attributable to officers despite their normally shorter stay on board. This finding can be explained by the high time pressure, a wide range of obligations and the high responsibility for personnel and material on board [39]. Improving the mental health of seafarers should therefore not be ignored by shipping companies and maritime stakeholders. Additionally, it is essential to make seafarers aware of the significance of this subject. It is proposed that measures should be designed for all seafarers with the aim of removing the taboo surrounding mental health [40].

Moreover, a program to improve mental health should be permanently offered and regularly promoted to the seafarers. Existing literature provides very limited evidence on preventive mental health interventions for seafarers. A recent systematic review identified only two intervention studies, both too heterogeneous to draw concrete recommendations for civilian maritime contexts; most documented programs were derived from military settings and may have limited applicability to merchant shipping [41]. Nevertheless, targeted preventive approaches exist, such as the “Wellness at Sea” program offered by the Sailors’ Society, which combines pre-departure training, online modules, and ongoing peer support to promote mental well-being among seafarers [42].

### Activity levels of seafarers

The overall mean activity level of seafarers (9,438 MET-min), as assessed by the GPAQ, was significantly higher than that of a reference group of 19,978 European adults from 28 EU countries (2,151 MET-min) [43]. Physical activity levels were significantly higher among ratings compared to officers (p = 0.005). The observed discrepancy in weekly MET-minutes between officers and ratings is substantial and likely rooted in the occupational demands aboard cargo vessels. These findings align with Oldenburg & Jensen (2019), who have shown higher physical activity levels among lower-ranked seafarers (777–801 kcal energy expenditure during work per day). This high activity is due to the nature of their work, which often involves manual labor and maintenance duties with limited rest. In contrast, officers typically engage in supervisory and administrative functions, which, while psychologically demanding, are generally less physically strenuous (568 kcal during work per day) [44].

The absence of a significant impact from leisure-time and travel-related activities emphasizes the dominance of work-related physical demands in this population. The self-reported nature of the GPAQ is prone to recall errors and social desirability biases, which may have led to an overestimation of physical activity levels. In particular, the “travel” domain appears questionable in the seafaring context, as seafarers reported relatively high values (805 MET-min/week) despite typically only short distances onboard; this may reflect an overestimation influenced by occasional, but irregular shore leave, which is not a weekly routine on most vessels [44]. Nevertheless, given the physically demanding nature of shipboard tasks, it is plausible that seafarers maintain a more active lifestyle compared to the land-based general population [43].

A comparison of the findings from the present study with those of a Thai population-based study from 2017 reveals that seafarers also exhibit significantly higher levels of physical activity during their leisure time (1,604 vs. 447 MET-min per week) [45]. This observation may be related to the limited availability of other leisure activities on board, which leads seafarers to using the ones available (e.g., gym) more often.

Significant differences in BMI were found between officers and ratings (p = 0.05). Ratings were more often of normal weight, while officers showed higher rates of overweight and obesity. This may be further influenced by the nature of the officers’ work with lower activity levels compared to ratings’ physically demanding work [44]. However, it should be acknowledged that nutrition is the key factor for body weight control. Neumann et al. (2021) showed that Asian seafarers often gain weight on board, because the diet is more like European’s high-calorie diet, characterized by large quantities of food, which can cause seafarers to overeat, especially when they are served food on board, that they are not used to eat at home. Asian seafarers are more likely to be overweight compared to those on land of their origin, while Europeans are more often overweight compared to Asians, whether on land or on board [46].

The high interest in sport competitions among both officers and ratings reflects a general motivation for physical activity on board. The slightly higher interest among officers may relate to their performance orientation and hierarchical career dynamics, where competitive environments are more familiar [47]. However, this difference lost significance after adjustment for culture and age, suggesting that occupational role alone does not fully explain motivational differences for sport competitions.

Interest in group and individual sports, as well as exercise instruction, showed no significant rank-related variation. This contrasts with previous findings, where ratings were more often interested in communal activities [20]. It is assumed that the officers’ increased interest in joint sports activities may be associated with the COVID-19 pandemic and the associated severe psychological stress and isolation. The qualitative approach from Carrera-Arce (2022) shows that physical activity and sport as well as social contacts were assessed as helpful by 19% of the participants during their shipboard stay during the pandemic [48].

The present study also identified several barriers to physical activities on board. As expected, physically demanding work was identified as a major barrier to physical activity in their leisure time among seafarers, with ratings significantly more affected than officers. The significant differences may be linked to the contrasting nature of labor. Beyond physical workload, educational status may further contribute to this disparity. Officers typically have higher formal education, which is associated with greater health awareness, self-efficacy, and physical activity engagement [18,49]. In contrast, lower education among ratings may limit access to health information and reduce motivation to engage in exercise. Time-related barriers were common, with 70.8% of participants reporting that their day is too busy for physical activity (officers vs. ratings: 44.9% vs. 30.7%, p = 0.001). Fatigue-related barriers were also prevalent, with 60.7% of participants feeling too tired after work. Taking people working on land as a reference group, white-collar workers also generally exhibit greater levels of physical activity during their leisure time in comparison with blue-collar workers [50].

Fear-related barriers, such as fear of injury, were more common among ratings (20.6% vs. 9.3%, p = 0.001), indicating a need for structured, accessible fitness programs that address both safety and competence particularly among lower ranks. These findings highlight the need for differentiated interventions that account for occupational workload and psychosocial barriers, rather than assuming uniform preferences.

### Health promotion

Interest in health promotion on board was generally high, with ratings showing significantly higher interest in health-related information compared to officers. The regression analysis revealed that age and culture were significant predictors of interest in health topics, with younger participants and those from non-European regions showing higher interest.

Given that ratings more frequently indicate barriers in health-promoting behavior, such as exercising in leisure time, higher interest in health-related topics in this target group is certainly desirable. Nevertheless, health promotion measures should not only be made attractive for ratings, but also for officers. The diverse starting points, expectations and socio-economic disparities, both between the ranks and due to cultural and age-related differences, demonstrate that the customized design of health promotion measures for seafarers on board cargo ships represents a complex challenge. Consequently, health promotion measures should not only be differentiated according to rank groups but should also consider various subject-related factors and address the individual needs of each crew member on board. This can be achieved through customized digital health promotion measures.

Health promotion programs can include components such as fitness activities, wellness programs, tracking health metrics, and providing access to telemedical services for remote workers on ships. Moreover, such a system should be able to provide tailored recommendations for action. Seafarers are a target group difficult to reach due to the unique nature of their employment. However, their poor physical accessibility could explain why they are so adept at using mobile devices [51]. These characteristics make seafarers the ideal target group for digital health promotion. By implementing digital health promotion, cargo shipping companies can enhance the overall health, safety and well-being of their employees. The establishment of such a system currently remains a prospective goal. However, the rapid development of AI-based assistance systems suggests that individualized health promotion, tailored to the specific needs of the user, may become reality in the near future. For example, the project AI-healthy ship [52] is an initiative that aims to promote health and well-being of seafarers on cargo ships under the use of AI-driven recommendations for health actions. Consequently, the undertaking of research and development projects in this domain is regarded as having a significant value.

### Strengths and limitations

The study has several strengths, including a large sample size and a high response rate (94.6%). Moreover, the research addresses seafarers on seagoing cargo ships, a population that is both underrepresented and difficult to access. The study thereby fills an important gap in health science-related literature and includes an innovative research topic. Standardized and validated questionnaires (WHO-5, PHQ-9 and GPAQ) were used, to ensure the reliability and validity of the findings.

However, some limitations must be noted. The cross-sectional design limits the ability to draw causal inferences. Women were rarely included, but this reflects the gender distribution in the target population. Data collection was limited to one shipping company, although the crew composition was multinational.

## Conclusion

This study provides valuable and novel comparative insights into the health and well-being among officers and ratings on merchant vessels. The findings highlight significant rank-based differences in well-being, physical activity, and interest in health promotion, suggesting that occupational role and associated work patterns may influence health behavior and status. Nevertheless, age and origin also emerged as determinants, with younger participants and those from Non-European regions showing more interest in supportive sport and health promotion programs. While existing literature consistently indicated increasing mental health risks among seafarers, studies about targeted preventive measures remain limited. These findings should therefore be considered in the development of health services.

To facilitate seafarers’ access to crucial health information and resources, digital health promotion measures, that integrate individualised approaches reflecting occupational, demographic, and cultural needs, should be established on vessels. Organizational commitment, such as formal policies, resource allocation and encouraging participation, will be essential for successful implementation. Future research should apply longitudinal and subgroup-focused designs to better understand causal relationships between occupational factors, demographic influence and health outcomes to facilitate the development of comprehensive, multilevel strategies to improve seafarer’s health and well-being.
